# Veterinary Education*Abstract of an Introductory Address delivered by Erskine S. Bates, M.D., D.V.S, on the occasion of the opening Fall Term, October, 1881.

**Published:** 1881-10

**Authors:** Erskine S. Bates


### ART. XVI.—VETERINARY EDUCATION.*

IT has long been the custon to open a course of medical lectures with a formal Introductory Address. In the opinion of many it is very beneficial to the college to assemble its friends—the alumni and students—together and in a social way discuss some subject in which all have an interest. The thoughts which I have imperfectly compiled for our present consideration are somewhat desultory, but in the main they bear especial reference to the subject of Veterinary Education.

---

* Abstract of an Introductory Address delivered by ERSKINE S. BATES, M.D., D.V.S , on the occasion of the opening Fall Term, October, 1881.

Several attempts have been made in this country to establish colleges or schools devoted to the science of comparative medicine and surgery, or what is more popularly known as veterinary medicine.

The first and earliest of which I can find any record was made by Robert Jennings, of Philadelphia. Dr. Jennings was not a graduate either of human or comparative medicine, although he was at that time and had been for many years a successful practitioner of the veterinary art. He had the reputation, however, of being well informed on medical subjects.

In the winter of 1846, during the sessions of the human medical schools, he gave a course of veterinary lectures in Philadelphia to a small class of medical students.

These lectures were continued thereafter for several years, or until the year 1850, when he conceived the idea of organizing a veterinary college. At first he received but little encouragement from those to whom he broached the subject, but finally, through the support of four human physicians, a doctor of divinity and several prominent citizens of Philadelphia, he succeeded in obtaining a charter for an institution called the Veterinary College of Philadelphia. The act granting this charter was approved April 15, 1852, nearly thirty years ago, and marks the date of the birth of the first veterinary college in America. This effort was evidently premature, for the college organization survived but a short time, dying with evidences of internal trouble and lack of pecuniary vitality.

About this period (1851) G. H. Dadd, of Boston, another self-made veterinary surgeon began publishing a veterinary journal. It flourished one year, experienced a severe collapse, finally was revived in 1855, and lived on for three years more. With the resuscitation of the journal in 1855, Mr. Dadd succeeded in obtaining a charter from the Massachusetts Legislature establishing the Boston Veterinary Institute. G. H. Dadd, C. M. Wood, R. Wood, and A. S. Copeman, (all practising veterinarians,) constituting the faculty and forming the editorial corps of the journal. The journal and institute both succumbed before completing the first dentition. The causes which led to this catastrophe do not plainly appear.

Dr. John Busteed, a human physician practising in this city, who had long been an enthusiastic student of comparative science, then tried his hand at the business of college making. In 1857 he succeeded in obtaining a charter by special act of the Legislature, establishing the college known as the New York College of Veterinary Surgeons. The doctor seems to have been a very persevering, earnest, enthusiastic man. Through his personal efforts sufficient funds were raised by private subscriptions to erect a handsome and commodious building on Twenty-third street, near Sixth avenue.

Dr. Busteed, assisted by two graduates in veterinary medicine, constituted the faculty at the opening of the college. For some reason they failed to serve the purpose when tried as teachers and lecturers. The college soon closed its doors and was afterwards sold and used for a livery stable. The building was finally destroyed by fire in 1865.

Nothing daunted by the failure of his first effort, Dr. Busteed continued to agitate the necessity and importance of having facilities in the United States for obtaining a knowledge of veterinary science.

In 1862 he procured an amendment to the old charter of 1857, and in 1864, two years later, having succeeded in organizing a new Board of Trustees, and forming a new faculty, he began another course of lectures.

The first term opened with one professor and three lecturers. Seven students matriculated during the term.

The history of this effort at establishing a veterinary college is a somewhat remarkable one.

During its first session, it counted seven students. In 1868, four years later, it had only one student during the entire term. In 1873, it had eighteen matriculants. The college suspended work, after an existence of ten years, at the close of the session of 1874–'75—the cause of its demise being internal disturbances, brought on, no doubt, by the perversity and peculiar character of its Board of Trustees.

In this connection, it is but just to say that Dr. John Busteed seems to be entitled, in every respect, to be called the father of veterinary medicine in this country. He seems to have done more than all others combined to agitate, popularize, and bring his subject before the people and the government of the country. Such enthusiasm, energy and perseverance as he carried into the cause, are worthy of the commendation, remembrance and imitation of all those who aspire to the title of D.V.S.

During the lifetime of the New York College of Veterinary Surgeons, the subject of veterinary education was being agitated in various parts of the country, and the discussion was bearing some fruit.

In 1862, the Ontario Veterinary College was organized, and the first course of lectures given in Toronto, Canada. This school is nominally under the supervision of the council of the Agricultural and Arts Association—Prof. Smith, a veterinary graduate of the Edinburgh school, being the Principal. From the opening lecture to the present time, the school has been a success. It has graduated 282 veterinary surgeons during its nineteen years of existence, and has a large class of native students.

In 1866, the Montreal Veterinary College was established as a veterinary department of the McGill University, under the direction of Dr. McEachran, F.R.C.V.S. This department has been in successful operation ever since its organization, having graduated about fifty students during the last fifteen years.

Between the years 1868–'73, the Illinois University, Cornell, Amherst, and Ohio Agricultural Colleges established veterinary departments. Later, the Maryland, Pennsylvania, Iowa, and Vermont State Agricultural Colleges tried to carry a veterinary course of lectures, as did also the Boston Institute. In tracing the history of these efforts, I have been forced to conclude that they all resulted in failure. At Cornell, even, the degree D.V.M. was conferred on only two students since the department was established.

A veterinary tail attached to an agricultural or literary kite seems to be destined to a very brief existence, or else is soon so overshadowed by its more popular associate that it quickly becomes too weak to be of any practical consequence. It seems to be a law of nature that the greater absorbs the less; the more popular gradually eclipsing the less attractive and unknown. Our whole educational history is filled with illustrations of this fact. Independent special schools grow strong and vigorous; departments and attachments of various kinds become beautifully less, and finally cease altogether, or exist only in name. Our law schools, and medical colleges, and institutes of divinity succeed and develop into strong, healthy seats of learning and science, just in proportion as they stand on a single broad, firm foundation, and are managed on the one idea principle, upheld and supported by teachers whose souls are in their work.

I stated that the N. Y. C. V. S. practically ceased to exist as a college in the Spring of 1875, or at the close of the term of 1874–'75.

The result of her demise was the birth of the American Veterinary College of New York, under the direction and management of Prof. Liautard. Born in April, 1875, the American college has continued its lectures up to the present time, graduating seventy-two D. V. S.'s during the six years it has been chartered as a college, and doing good work in the cause of veterinary education.

In 1877 an effort was made by the trustees to again revive the New York College of Veterinary Surgeons. A Faculty was constituted and one course of lectures delivered to a respectable number of students. The course of the trustees, however, again created dissatisfaction, and the Faculty, realizing that they could no longer retain their self-respect and continue their labors, resigned in a body. To-day, the N. Y. C. V. S. is a wreck, existing only in name, her halls deserted, her laboratory, museum and apparatus destroyed or scattered beyond redemption. Her final convulsion brought into existence our own Columbia.

Youngest of all the veterinary family, she aspires to be the leading authority in all that pertains to the advancement of veterinary education, and the development of our knowledge of comparative medicine. Established on a broad and liberal basis, she stands on her own merits, has implicit faith in the ability of her

Faculty to teach the most advanced principles of veterinary medicine and surgery, points with pride to the quality and character of her alumni, and takes the deepest interest in all that relates to the progress of her pupils. Such, in brief, is the history of veterinary medicine up to the present time.

Let us now for a moment consider what has been accomplished, and learn the status of our present condition.

In the first place, whatever has been done has been accomplished by personal individual effort.

Government, which in England, France and Germany is exceedingly liberal and generous in the support of veterinary science, has done nothing for us. In no practical form has there been even sympathy or encouragement manifested outside of the veterinary and human medical professions. If I mistake not, in every instance, with a single exception, those who acted as the Faculty of the colleges to which I have referred had to assume the pecuniary burdens. All honor to such pioneers as Jennings, Dadd, Busteed and their colleagues. They paved the way and made it easier for those who have come after them.

Less than a quarter of a century ago veterinarians and veterinary science were almost an unknown quantity. To-day we have in Canada and the United States four schools or colleges in successful operation, with a total attendance of a little over three hundred pupils. Interested in teaching and raising the standard of veterinary education are forty-three human and comparative physicians, many of them men with a national and trans-Atlantic reputation, all of them recognized in the world of science for their high standing and professional attainments.

There are three journals (two monthlies and one quarterly) published in this city devoted to the advancement of comparative medical science and the interests of the veterinary art, and I think I do not exaggerate the facts when I say that they are rapidly approaching in general character and quality the best journals of the same kind published across the water. It is not much over fifty years since we could boast of having a really well-educated veterinarian within our borders. To-day there are between eight hundred and a thousand men engaged in the practice of veterinary profession—probably a little over eight hundred all told. This number is composed of regular graduates of the veterinary school, of human physicians, self-educated veterinarians, licentiates and quacks. The graduated human and comparative physicians number about five hundred—over half of the entire number.

The self-educated constitute a small minority, probably not over twenty at most. By the term self-educated I refer to a class of intelligent men who long ago, seeing a lucrative business in veterinary practice, armed themselves with a library and diligently studied it until they had practically mastered all that was known on the subject.

Such men always command my respect, and I never fail to extend to them the right hand of fellowship.

The licentiates are those who have been examined by the censors of the New York County Veterinary Medical Society, and have been found sufficiently well-informed to be granted a license to practice in the county in which that society has jurisdiction. The number of licentiates is about forty.

Deducting these men and the graduated veterinarians from our total number, and we have left quite an army of irregular practitioners or quacks. By the term quack, I mean men who have not the first idea of what constitutes a diseased condition or how to treat it. They fire and blister and bleed and cut and drench and dose, regardless of the pain, torture, deformity or death that may follow. Such men not only injure and destroy the animals unfortunate enough to fall under their care, but they injure seriously the reputation and business of those who are thoroughly competent and properly qualified by years of training, close observation and hard study.

In the present dearth of good veterinarians there is no remedy for this evil. We can only work and wait, hoping that in the onward march of veterinary medicine the quack brigade will become extinct.

Our most useful and valuable animals are now less often subjected to uncalled-for medication and cruel surgical operations than heretofore, and they will be still less so as the number of competent veterinarians increases.

Just in proportion as this change takes place, will the importance of veterinary science be more and more esteemed, and its worthy practitioners respected. Even now the line is being sharply defined between the regular and irregular practitioner—only the graduates of the schools being recognized as sufficiently competent to give evidence as experts in the United States Courts, and to receive appointments in the army.

Twenty-five years ago, even in Europe, the veterinary profession was a most humble one, and recruited from the very lowest of the educated classes. Scarcely any of its members were received into decent society, and certainly none could ever hope to rise to any social eminence. What a contrast we have in the present status of the veterinary practitioner!

The profession has been raised out of the dirt; the curriculum of the veterinary colleges has been extended and made more liberal; the examinations are searching, and the diplomas only given to such as are thoroughly qualified. The British Parliament has recently enacted a bill forbidding any one to assume or use the title of veterinary surgeon, or to practice veterinary medicine and surgery except he has a diploma or license from the proper authorities. Their social standing (a point on which Englishmen are often sensitive) has been materially raised. I read, not long since, that the Prince of Wales had invited an English veterinarian to dine with him, in order to give social recognition to the veterinary profession.

In the United States, the social position of veterinarians does not depend on the recognition of prince or potentate, but on the kind of training our young men receive and the character and reputation they develop in their professional life. If properly educated, they will continually add to the dignity and distinction of the profession, eventually placing it among those most cultivated by gifted minds.

This feature is well illustrated by an incident in the late International Medical Congress. Prof. Pasteur read an address on Animal Vaccination which created the deepest interest of any paper read before that august body. It not only affected profoundly the medical savants before whom it was read, but it occasioned wide-spread discussion in the agricultural and scientific world. At its close, Sir James Paget thanked him in the name of the International Medical Congress and the English people, explaining that he had done and was doing for the lower animals just what Jenner had done for the human family. Pasteur's theories if they bear the test of practical application, certainly promise to reduce to a minimum the contagious diseases of animals and introduce us to a prophylactic agency of the utmost importance to both comparative and human medicine. Veterinary science thus scores another triumph in the cause of human progress. It is conceded that the most important discoveries in human medical science have originated, directly or indirectly, from observations on animals. In my opinion, there is very little doubt that it is in comparative medicine that we are still to find our grandest fields for original investigation and research. Let us hope that some who are listening here this evening will yet surprise the nations with the importance and extent of their discoveries in veterinary medicine. Bear in mind, however, that such tasks and results require gifts of a high order as well as the most rigid and thorough medical training.

In my opinion, two things have done more than all other influences combined to push medicine forward as a science during the last fifty years, during which time the real advance that has been made eclipses all that had been previously known.

These two things are, first, the study of the gross and minute lesions of the body in connection with well-applied logic as to the real nature of morbid actions; secondly, experimental pathology and therapeutics. By experimental pathology I mean well-considered operations upon the lower animals with a view to studying the *modus operandi* of morbid force and the result it produces.

Experimental therapeutics is part and parcel of experimental pathology, advancing hand in hand with it. The two are inseparably connected. For we no sooner discern the nature and extent of a morbid process than we are eager to set in operation some antagonizing agency to counteract it.

Our present therapeutical advances are almost wholly based on the observations which have been made on animals in diseased and healthy conditions. To the veterinarian properly belongs the investigation of diseases in the lower animals.

Who is so well qualified as he to observe, collect and digest the effect of drugs in controlling such disease?

I assure you, gentlemen, who are just beginning, and those already engaged in the study of this noble and important profession, that there is no department of medical science possessing such broad and untrodden fields, so rich in future promise as those to which I have just referred.

Much as we have lately discovered concerning the diseases of animals and their relation to the human family, much more is still to be learned, and each item of information gleaned will be of positive pecuniary advantage both to the discoverer and the country at large.

Let me illustrate the pecuniary point by referring again to Prof. Pasteur's germ theory. France loses every year by splenic fever animals to the value of 20,000,000 francs. If his researches and experiment bear the test of practical application the total 20,000,000 francs will be saved to that country. Again, in Holland the dreaded pleuro-pneumonia has been kept under complete control by the veterinary corps. How? By innoculation and isolation; comparative medicine thus saving that country from immense losses in valuable property.

When viewed in connection with such results, the veterinarian assumes a position of the first magnitude, and his professional work becomes of the highest importance.

Most of the enlightened nations, except our own, appreciate such labors at their full value. France annually appropriates 750,000 francs towards the support of veterinary education. She has a graduated veterinarian for every 1,100 head of her equine property. Germany has 1,300 graduated veterinary surgeons, one to every 1,500 horses. Even Egypt manifests a commendable interest in the welfare of her domestic animals. The United States has one graduated veterinarian for every 200,000 of her domestic animals.

The necessity for recognizing the existence and utility of veterinary sanitary science was never greater in this country than it is to-day. It is to the increasing herds in the great West that not only England, but a large proportion of the people of Europe, must, to a considerable extent, look for their animal food in the immediate future. If the countless herds roaming the great western plains of our vast continent are to be protected and preserved from the ravages of the great animal plagues, veterinary science must furnish the skill and numbers to do it.

There is another branch of veterinary and human medical education which has made in the past few years, and is still making, great advances—I allude to the art of preserving health, prophylactic medicine, sanitary science.

The importance of these subjects has gained greatly in popular estimation. Public opinion realizes that it is more economical, as well as more scientific, to prevent disease than it is to cure it. Sanitary veterinary education, although yet in its infancy, covers a broad domain of useful importance.

It is in this field that the comparative physician is destined to achieve the most marked distinction. National, state, city and town Health Boards are forming all over the country. Without the services of a well-educated veterinarian they are deprived of half their strength and usefulness.

It is a pretty well established fact that a large proportion of the contagious and infectious diseases are preventable in human beings as well as in the lower animals. There is also no longer any question that many of the diseases to which mankind is subject are directly traceable to diseased conditions existing in some of the domestic animals which furnish our food supplies.

This truth is of especial importance to the American public, for we are an eminently meat-eating people. Let me illustrate this point. In England seventy-one of the recent epidemics were traced directly to an infected milk supply. Fifty of them were epidemics of typhoid fever, and caused the sickness of 3,500 people. Scarlatina scored 14, and counted 800 victims; diphtheria 7, and afflicted 500 victims. It is estimated that a tuberculous milk supply will tubercularize 50 per cent. of the children unfortunate enough to feed upon it. If that source is to be cut off and disease to be prevented, the Veterinary Sanitary Inspector becomes an important factor in the work. A veterinarian should not only be able to treat and cure the diseases of animals, but he should be thoroughly familiar with, and quick to detect, all the pathological conditions of diseased flesh.

The educating of young men so that they are competent to inspect all articles of animal food thus becomes of very great importance to us as a people. Not only the individual health of the nation is involved, but vast moneyed interests are at stake.

Within the last year great consternation and discussion followed when England scheduled our cattle, and Germany and the Latin nations put an embargo on the importation of pork from the United States. I believe that those governments were justified in such action. The cheapness and abundance which abound in this country promote the evils of which they complained.

When the diseased meat of cows, calves, sheep and swine can be bought for one-half or two cents per pound and made up into bologna sausages which wholesale at ten and retail at fifteen or twenty cents per pound, there will be plenty of men engaged in a business so lucrative, regardless of consequences. We have been quietly investigating this subject during the past two years, and have had specimens forwarded to us secretly from the manufactories, which, on examination, proved to be the livers of cattle ulcerated to such an extent that the pus could be squeezed out of them like honey out of the honeycomb. Huge cancerous masses have fallen in our way. Kidneys in advanced stages of suppuration and various portions of the body which were literally honeycombed with vast numbers of parasites.

In these manufactories—and they are generally carried on under cover—nothing is wasted, and it would seem the greater the amount of disease the more succulent and juicy the material prepared from it.

Even the old, worn-out and diseased horses are eagerly purchased, and in the form of equine stew, made to furnish a valuable material for fattening hogs.

In view of some such facts as these which have fallen under my observation, it will not seem strange that unusual maladies are directly traceable to our animal food, or that contagious and strangely fatal diseases are developed in various sections of our own country.

Do not understand me as saying that this condition of things is general. What I do say is, that I have learned enough from my observations to declare positively that there is sufficient leaven in this subject to leaven the whole lump. This matter is assuming such an important and formidable character that the inspection of our animals should be of the most rigid character, both before and after slaughter.

This is a subject that directly affects our export trade; a matter of no small moment to us as a people. The exporting of live stock and animal products involves immense results, and bids fair to become of more importance than the export trade of King Cotton. During the year 1880 there were exported 83,434 hogs; 18,756 horned cattle; 3,060 horses; 5,198 mules; 209,137 sheep, besides fowls, etc., at a total valuation of $15,882,120. The value of animal products exported during the same year was $145,251,256. Total of live stock and animal products, $161,140,376. The value of cotton exported and also produced during the same time amounted to $221,517,323.

If the present increase continues our exports in live stock and animal products will, in a few years, exceed those of the cotton interests. Such statistics preach the most effective sermons. They tell us, in most unmistakble terms, that veterinary sanitary inspection should be more general than it is; that it should be extended to every section of the country, and that it must be done if we are to meet and suppress the gigantic losses and evils which now threaten us through diseased animals.

To my mind the first step that should be taken in meeting this condition of things, should be the educating of our young men thoroughly and well in the science of Comparative Medicine and Surgery, and especially in the department of Comparative Pathology, or a knowledge of the effects of disease, immediate and remote, on animal flesh. All of us are directly or indirectly thus interested in this question of veterinary education. I have no doubt that in the near future this question will receive from the general public the attention and recognition that its importance demands. A country with such extensive and far-reaching moneyed interests at stake; a country which can produce an Iroquois, a Maud S., or whose cattle are represented by such queens of the bovine family as Jersey Belle, will, ere long, have a veterinary army which in numbers, general intelligence, professional skill, and gentlemanly bearing, will challenge the world.

Gentlemen, many of you are listening this evening to your first lecture in your veterinary education. Let me impress on your minds that the profession of comparative medicine and surgery is not one whit less humane, noble and important than its sister science, human medicine. Human physicians everywhere recognize the great value of trained skill and professional knowledge which is included in the terms veterinary science and graduated veterinarian. They welcome the veterinary physician and surgeon as a brother practitioner, engaged in the same grand crusade against pain, disease and death, and they will extend to you such professional courtesy and social recognition as your knowledge and general reputation command. See to it, then, that you so improve your time and opportunities that when you graduate from these halls, that by your scientific attainments and gentlemanly bearing you are fully equipped and prepared to join forces or take honorable issue in the progress of a common warfare. Your development here, your student life is the embryo of your future career. Such habits as you form and fix here will make or mar your success hereafter. So far as you yourselves are concerned, you cannot overestimate the importance of the work you accomplish. To secure the greatest benefit from your instruction, you must early learn to make haste slowly and steadily. Do not be over anxious to get clinical knowledge. I know how much more interesting and exciting it is to see a case of lively colic than it is to read about it.

Of late years we have been inclined to overdo this matter of clinical teaching, and we are in great danger of turning out a class of graduaes who are litttle, if any better than the grooms and superannuated stablemen who claim to be so skilled in treating animals, and whose whole knowledge is based on the cases they have seen.

Learn the principles of your profession first and make the practical application follow. Post up thoroughly in anatomy, physiology, histology, pathology, chemistry, microscopy, etc., before you attempt a differential diagnosis between an ulcerated schneidenan membrane and a doubtful case of glanders. Too much clinical instruction and too little didactic teaching tends to make professional dwarfs.

Carefully fixing in the mind the most important principles and facts of veteninary science, and crowning the work with practical observations, cannot fail to make you giants in your profession. The power to observe and investigate things accurately is of primary importance. An uncultivated person is very apt to think it is an easy matter to make a correct observation or a careful investigation. It is, however, extremely difficult. Observation requires peculiar skill and great attention.

So many things are to be considered, and there are so many sources of fallacy, that comparatively few men are capable of such work, and as a consequence very few complete observations have been collected. In veterinary science every student should educate all his senses, and especially train himself in the habit of making careful and accurate observation.

All who have studied medicine are, I believe, prepared to admit that the education of a professional man is never completed. No one has ever mastered all the phenomena of disease, and no one ever will. There is always some new truth to learn, some new complication arising to tax our skill and test our ingenuity. Students are very apt to forget this fact, and appear oftentimes to conclude that they are sufficiently educated when they have attended a few lectures and demonstrations, when they have seen a few cases in the infirmary and obtained the requisite number of signatures to a diploma. It should be your ambition, as it certainly is your duty, to obtain all the knowledge and skill possible during your sojourn here. Every day should find you better fitted for the prevention and cure of disease, but you should accept as a cardinal fact that one of the most important results of your college course will be the mental training which teaches you how to learn. While gathering and memorizings facts and principles here you are becoming disciplined in habits of study which will be of the greatest possible benefit hereafter.

Veterinary science offers you great opportunities and grand possibilities, but to make a success in it brains are needed, plenty of brains, and they must be well cultivated too. Thick heads and thin skulls may survive the intricacies of theology or the subtilities of law. The boy who is fit for nothing else may do for a human physician, but he will make a dead failure as a veterinarian.

But I forbear. The limits to which I am assigned will not permit me to continue this subject further.

On behalf of the faculty I extend kindly greeting to those who have returned to us for a final struggle with the knotty and difficult problems of veterinary science, and at the same time I welcome our new friends to a term of earnest labor and profitable acquaintance with the curriculum of Columbia Veterinary College.

